# Developing a machine learning model with enhanced performance for predicting COVID‐19 from patients presenting to the emergency room with acute respiratory symptoms

**DOI:** 10.1049/syb2.12101

**Published:** 2024-10-29

**Authors:** Maha Mesfer Alghamdi, Naael H. Alazwary, Waleed A. Alsowayan, Mohmmed Algamdi, Ahmed F. Alohali, Mustafa A. Yasawy, Abeer M. Alghamdi, Abdullah M. Alassaf, Mohammed R. Alshehri, Hussein A. Aljaziri, Nujoud H. Almoqati, Shatha S. Alghamdi, Norah A. Bin Magbel, Tareq A. AlMazeedi, Nashaat K. Neyazi, Mona M. Alghamdi, Mohammed N. Alazwary

**Affiliations:** ^1^ College of Applied Studies and Community Service, Department of Computer Science and Engineering King Saud University Riyadh Saudi Arabia; ^2^ Department of Internal Medicine Security Forces Hospital Riyadh Saudi Arabia; ^3^ Critical Care Service Administration King Fahad Medical City Riyadh Saudi Arabia; ^4^ Department of Cardiac Surgery‐King Salman Heart Centre King Fahad Medical City Riyadh Saudi Arabia; ^5^ Department of General Surgery Ministry of National Guard Health Riyadh Saudi Arabia; ^6^ Aviation Medical Center Aviation Medicine Riyadh Saudi Arabia

**Keywords:** big data, biomedical engineering, ontologies (artificial intelligence)

## Abstract

Artificial Intelligence is playing a crucial role in healthcare by enhancing decision‐making and data analysis, particularly during the COVID‐19 pandemic. This virus affects individuals across all age groups, but its impact is more severe on the elderly and those with underlying health issues like chronic diseases. This study aimed to develop a machine learning model to improve the prediction of COVID‐19 in patients with acute respiratory symptoms. Data from 915 patients in two hospitals in Saudi Arabia were used, categorized into four groups based on chronic lung conditions and COVID‐19 status. Four supervised machine learning algorithms—Random Forest, Bagging classifier, Decision Tree, and Logistic Regression—were employed to predict COVID‐19. Feature selection identified 12 key variables for prediction, including CXR abnormalities, smoking status, and WBC count. The Random Forest model showed the highest accuracy at 99.07%, followed by Decision Tree, Bagging classifier, and Logistic Regression. The study concluded that machine learning algorithms, particularly Random Forest, can effectively predict and classify COVID‐19 cases, supporting the development of computer‐assisted diagnostic tools in healthcare.

## Graphical abstract

The study focused on developing a reliable machine learning model for the prediction of COVID‐19 from patients with acute respiratory symptoms. The Random Forest produced the highest results.

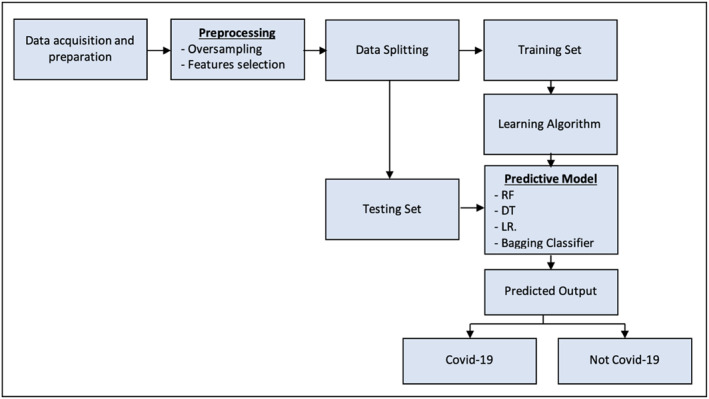

## 1 INTRODUCTION

The novel coronavirus (COVID‐19) which emerged in Wuhan, a Chinese city in December 2019, resulted in one of the worst global health problems in modern times [1, 2]. It is now known that the virus spreads easily through the respiratory tract when a healthy individual interacts with an infected person. It is highly infectious, and it spreads rapidly across the globe causing catastrophic health problems as well as crippling down the economies and other development activities [3, 4]. The COVID‐19 was declared a pandemic by the World Health Organisation (WHO) soon after it was identified in different countries [5, 6, 7, 8]. In response, governments across the globe put in place several containment measures to mitigate the spread of the virus including infection detection tests, social distancing, and lockdowns which impacted on different aspects of life [9]. However, as argued by Rodriguez et al. [3], the emergence of new strains of COVID‐19 and people's negative behaviours such as refusal to be vaccinated, make it hard to eradicate the disease and this is becoming a seasonal disease.

The virus presents different symptoms to individuals including cough, fever, sore throat, headache, exhaustion, and discomfort in muscles, and this constitutes one of its main problems [10]. Individuals with mild to moderate COVID‐19 experience dry cough, whereas individuals with severe COVID‐19 experience fatigue, fever and shortness of breath (dyspnoea). When COVID‐19 affects the lungs, pneumonia may emerge, which may lead to severe and critical illness [11, 12, 13]. The elderly and people with certain underlying health conditions, such as asthma, diabetes and heart diseases are more susceptible to severe illness [14, 15, 16, 17]. In addition to the serious implications including deaths, COVID‐19 also has serious negative impact on mental health in the community as well as that of healthcare staff [18].

Across the world, it is known that chronic illnesses are the major cause of death and disability [19, 20]. Health conditions such as hypertension, asthma, diabetes, cancer, and chronic obstructive pulmonary disease (COPD) are ranked among the top causes of death [21, 22]. The negative consequences of COVID‐19 infection led to several research to be undertaken to develop reliable models that can be used by the medical staff for the early diagnosis of COVID‐19. One major benefit of early diagnosis of COVID‐19 is that life can be saved if someone receives medical treatment early on. Some innovative solutions from computer science such as image processing and bioinformatics have been developed by embracing Artificial Intelligence (AI) and Machine Learning (ML) applications in healthcare contexts [23]. In addition to supporting some of the clinical procedures, ML can also be used in the process of identifying COVID‐19 utilising image and textual data. The downside of ML is that it requires a huge amount of data for the classification and prediction of diseases. Supervised machine learning algorithms require annotated data to be able to classify the text or images into separate groups. However, there has been significant progress in the past decade in terms of using ML to address some of the critical projects in health settings. For instance, the recent pandemic has brought many researchers from across the globe to focus on resolving the problem at hand. Several studies, as demonstrated in the literature review section, have been conducted to demonstrate the application of ML and deep learning (DL) techniques in the identification of COVID‐19.

An analysis of the previous studies reveals some inconsistencies given the wide array of methods used, images and a wide variety of data [6, 7, 8]. For instance, it was clear that the current performance of the models was inadequate. One of the most obvious weaknesses of the studies is the use of relatively low number of cases. Yet, the amount and quality of data is critical when developing a reliable model for the detection and prediction of COVID‐19. The data that were generated during the pandemic were aggregated from different sources in varied formats and were often not of high quality. Therefore, it is possible that the datasets that were used in the initial studies were constructed hurriedly and lacked the quality and precision that one would ideally like. Given the nature of the datasets, it is possible that the use of incomplete and poor‐quality datasets resulted in the development of inaccurate predictive models that affect their use for effective policy decision making. Most of the studies that were consulted relied on the use of limited datasets. We, therefore, consider that the use of multi‐datasets or hybrid datasets which consist of clinical, mammographic and demographic data could generate more credible models given that each dataset has significant factors capable of representing the true identity of the infected patients, and the subsequent deployment of the model in the real world. To achieve more accurate and precise prediction models, it is important for the patient's history of other existing ailments such as diabetes, heart, liver or other chronic conditions along with their age and gender to be taken into account. The significance of these factors influences the characteristics of COVID‐19 immensely for specific patients compared with other pneumonia diseases that are known to date. The other key observation is the lack of understanding of epidemiological theory, beliefs and knowhow of many AI researchers. This results in situations where many of the AI‐based systems are created without input from medical professionals, hence, limiting their usefulness and reliability in practice. To this end, it is important that the development of any implemented AI‐based system should aim to involve medical experts in the design and implementation.

Our paper presents a significant contribution in the field of ML by developing an advanced model that leverages pre‐trained models to achieve enhanced performance. This approach was applied to medical data of patients presenting to the emergency room with acute respiratory problems. The data include demographics, respiratory symptoms, comorbidities, information about the current CXR images and laboratory test results derived from 915 patients from two hospitals in Riyadh, Saudi Arabia. The two hospitals are big hospitals in the country and the data produced are reliable for the evaluation of the new model. The data were used to design four supervised Machine Learning Algorithms including Random Rorest (RF), Decision Tree (DT), Logistics Regression (LR), and Bagging Classifier. These supervised machine learning algorithms were used to predict COVID‐19 infection using medical analysis data from patients presenting to the emergency room with acute respiratory symptoms. To prevent the problem of overfitting and to enhance the performance of the algorithms, the random oversampling technique was employed which helped to increase the non‐COVID‐19 samples. Prior to the training process, the feature selection technique was applied to identify the 12 key features for the prediction process. Our proposed models can be used across the world for effective screening and prioritisation of testing of COVID‐19 among the general population. In addition, our study involved working collaboratively with specialist doctors in intensive care units and the respiratory department who guided us throughout the study in terms of what they felt was required for effective patient management. Again, this addresses the weakness cited above regarding the development of models by researchers without any input from specialist medical doctors. In this case, our study demonstrates the importance of interdisciplinary collaborations between the researchers and the medical practitioners. This collaboration holds great promise for advancing the field of healthcare and optimising patient care in critical settings.

## 2 LITERATURE REVIEW

This section presents several studies carried out across the world in search of a reliable and cost‐effective model for the identification of COVID‐19 to combat the pandemic. This includes some recent studies which discuss some of the machine learning models being used today. For example: In a study by Yao et al. [24], an investigation into the detection of severely ill patients with COVID‐19 from those with mild symptoms using the clinical information and the blood/urine test data was conducted. They made use of the Support Vector Machine (SVM) model for detecting and classifying COVID‐19 cases. The clinical information included age, sex, body temperature, heart rate, respiratory rate and blood pressure. In their study, the SVM demonstrated a highly promising accuracy for the detection of 32 features that are linked with the COVID‐19 severity. The 32 features were screened further to identify inter‐feature redundancy. Finally, the SVM model was trained using 28 features and the test results revealed the effectiveness of the model by achieving an accuracy of 81.48%, sensitivity of 83.33% and specificity of 100%.

In the same vein, Brinati et al. [25] proposed two machine learning models namely Logistic Regression and Random Forest to distinguish between patients who are either COVID‐19 positive or negative using haematochemical values from routine blood. The study involved working with 279 patients admitted at the San Raffaele Hospital in Milan, Italy. The patients presented themselves to the Emergency Room with COVID‐19 symptoms. A few basic demographics including gender and age as well as a small array of routine blood tests (white blood cells counts, and the platelets, CRP, AST, ALT, GGT, ALP, LDH plasma levels) were captured. The number of occurrences for the negative and positive class was 102 (37%) and 177 (63%), respectively. In this case, the dataset was imbalanced in favour of the positive cases. They developed a simple decision aid called an interpretable decision tree model for clinicians to use for interpreting blood tests for suspected COVID‐19 cases. The two models achieved sensitivity rate between 92% and 95%, and accuracy of between 82% and 86%.

In another study conducted by Yang et al. [26], four machine learning models (Logistic regression, Decision tree, Random Forest, Gradient boosting decision tree (GBDT)) were developed. These were able to incorporate patient demographic features, such as age, sex, and race with 27 routine laboratory tests to predict an individual's covid infection status. This data was generated from the New York Presbyterian Hospital and Weill Cornell Medicine (NYPH/WCM) to select and predict an individual's COVID‐19 infection status. A gradient boosting decision tree (GBDT) was trained using laboratory results obtained within 2 days before the release of COVID‐19's RT‐PCR results. From 3356 COVID‐19 RT‐PCR tested patients, 1402 positive and 1954 negative and these tests were evaluated at a metropolitan hospital. The efficiency of the GBDT model was demonstrated by the experimental results which achieved the best performance: sensitivity 76.10%, specificity 80.80%, and AUC of 85.40%.

Similarly, Hu et al. [27] developed predictive models using data from 183 patients who were admitted with severe COVID‐19 infection. Of these, 115 were survivors and 68 were non‐survivors from the Sino‐French New City Branch of Tongji Hospital in Wuhan. Machine learning techniques were used to select the features and make prediction of the patients' outcomes. They selected four variables including age, hsCRP, lymphocyte count and d‐dimer and they proposed five models including Logistic Regression, Random Forest, Partial Least Squares Regression (PLSR), Elastic Net and Bagged Flexible Discriminant Analysis (BFDA) for the diagnosis of and prediction of the seriousness of COVID‐19 patients. The obtained results demonstrated that the logistic regression model outperforms other machine learning models by achieving a sensitivity of 89.20%, specificity of 68.70% and AUC of 89.20%. The limitation of the study is the limited number of cases used.

Khanday et al. [28] employed 212 clinical reports labelled in four classes namely COVID, SARS, ARDS, and both COVID and ARDS. Techniques such as Term frequency/inverse document frequency (TF/IDF), Bag of Words (BOW) and report length were used to perform feature engineering. The machine learning algorithms are useful for the classification of clinical reports into four different classes. Based on the classification results, it was revealed that logistic regression and multinomial Naaive Bayesian classifier gives excellent results including 94% precision, 96% recall, 95% F1‐score and 96.2% accuracy. In yet another study by Sun et al. [29], 336 cases of patients infected COVID‐19 in Shanghai were retrospectively enrolled and divided into training and test datasets. On top of this, 220 clinical and laboratory observations/records were collected. More than 200 clinical and laboratory features were analysed and this led to the proposal of an SVM based model to predict the opportunity of patients' progress into severe/critical symptoms. A total of 26 of these features were found to be statistically significantly associated with the clinical outcome (which could be severe or critical symptom) of these COVID‐19 infected patients. Interestingly, despite 10 out of 26 patients having severe/critical symptoms and 16 with mild symptoms when they all underwent clinical and laboratory examinations, it emerged that the features of all the cases were similar. The model produced an accuracy rate of 77.5% and specificity of 78.4%.

As evidenced by the examples of studies cited above, it is clear that several studies have been conducted to explore the development of reliable methods for the detection and prediction of COVID‐19. Table 1 below provides a summary of some of the studies that were consulted prior to the conduct of the study. This includes methods used as well as the evaluation of the effectiveness of the different models.

**TABLE 1:** A summary of models used for the detection and prediction of COVID‐19.

Study	Features	Sample size	Number of feature	Objective	Techniques	Performance (%)
Yao et al. [24]	Clinical information and blood/urine test data	137 (75 severely ill COVID‐19 infected patients 62 patients with mild symptoms)	28	Detect the COVID‐19 severely ill patients from those with only mild symptoms	Support vector machine	Accuracy = 81.48
						Sensitivity = 83.33
						Specificity = 100
Brinati et al. [25]	Routine blood exams (white blood cells counts, and the platelets, CRP, AST, ALT, GGT, ALP, LDH plasma levels)	279 (177 COVID‐19 102 non‐COVID‐19)	13	Detect COVID‐19 from non‐COVID‐19	‐ Logistic regression	Accuracy 86
					‐ Random forest	Sensitivity 95
Yang et al. [26]	Demographic features with routine laboratory	3356 (1402 positive 1954 negative)	30	Detect COVID‐19 from non‐COVID‐19	‐ Logistic regression	Sensitivity = 76.1
					‐ Decision tree	Specificity = 80.8
					‐ Random forest	AUC = 85.40
					‐ Gradient boosting decision tree (GBDT)	
Hu et al. [27]	Demographic, clinical and first laboratory findings	183 (115 COVID‐19 68 non‐COVID‐19)	4	Detect COVID‐19 from non‐COVID‐19	‐ Logistic regression	Sensitivity = 89.2
					‐ Random forest	Specificity = 68.70
					‐ PLSR	AUC = 89.20
					‐ Elastic net	
					‐ BFDA	
Khanday et al. [28]	Clinical reports	212 clinical reports		Multi classification	‐ Logistic regression	Precision = 94
						Recall = 96
					‐ Multinomial Naaive Bayesian	F1‐score = 95
						Accuracy = 96.2%
Sun et al. [29]	Clinical and laboratory features	−336 COVID‐19patients	36	Prediction model for severe/critical symptom	Support vector machine	Accuracy: 77.5%
						Specificity: 78.4%

### 2.1 Summary and contribution of our study

The literature review demonstrated that the models that were developed presented some limitations. For instance, it was clear that the current performance of the models was inadequate. One of the most obvious weaknesses of the studies is the use of relatively low number of cases. The amount and quality of data is critical when developing a reliable model for the detection and prediction of COVID‐19. The data that were generated during the pandemic were aggregated from different sources in varied formats and were often not of high quality. Therefore, it is possible that the datasets that were used in the initial studies were constructed hurriedly and lacked the quality and precision that one would ideally like. Given the nature of the datasets, it is possible that the use of incomplete and poor‐quality datasets resulted in the development of inaccurate predictive models that affect their use for effective policy decision making. Most of the studies that were consulted relied on the use of limited datasets.

We, therefore, consider that the use of multi‐datasets or hybrid datasets which consist of clinical, mammographic and demographic data could generate more credible models given that each dataset has a significant factor capable of representing the true identity of the infected patients, and the subsequent deployment of the model in the real world. To achieve more accurate and precise prediction models, it is important for the patient's history of other existing ailments, such as diabetes, heart, liver or other chronic conditions along with their age and gender to be taken into account. The significance of these factors influences the characteristics of COVID‐19 immensely for specific patients compared with other pneumonia diseases that are known to date. The other key observation is the lack of understanding of epidemiological theory, beliefs, and knowhow of many AI researchers. This results in situations where many of the AI‐based systems are created without input from medical professionals, hence, limiting their usefulness and reliability in practice. To this end, it is important that the development of any implemented AI‐based system should aim to involve medical experts in the design and implementation.

It is against this background that our study experiment is likely to make a significant contribution to the existing body of knowledge and practice. We prepared a dataset from two hospitals in Saudi Arabia working in partnership with medical staff. The two hospitals are big hospitals in the country and the data produced are reliable for the evaluation of the new models. The medical staff's expertise helped us to know the important data that we needed to build a method that facilitates the detection of COVID‐19. We noticed that the data and the features available in literature were not suitable and not enough, hence, we collected data with many important features with the guidance of the specialist medical doctors. To achieve more accurate and precise prediction models, it is important for the patient's history of other existing ailments, such as diabetes, heart, liver or other chronic conditions along with their age and gender to be taken into account. As a result, we held meetings and discussions with specialist medical doctors and went ahead to collect patient demographics, respiratory symptoms, comorbidities, CXR images with CXR information, laboratory work and PCR test results. This produced a total of 36 features in the dataset that were found to be helpful in detecting COVID‐19.

ML techniques were used to identify the features and for predicting the patients' outcomes. The main contribution of our study is how it leveraged pre‐trained models to develop enhanced models for the prediction of COVID‐19.

Machine learning models vary in their performance depending on the algorithm used. Some algorithms are suitable for small data sets, which is the case for our data. Therefore, we chose an algorithm that can handle small data well. We trained several models using this algorithm and selected the one that had the best results, took the least time to train, and used the features that were relevant for Covid diagnosis by specialist doctors.

## 3 RESEARCH METHODOLOGY

The paper presents a four‐step method to predict COVID‐19 using machine learning. The steps are:

1. Data acquisition: The study describes how the datasets for the study were obtained and analysed.
2. Data pre‐processing: This is one of the critical stages in the study as it contains details of data pre‐processing. The study applies a random oversampling technique to balance the classes and prevent overfitting.
3. Training and prediction: The study uses four supervised machine learning algorithms, namely Random Forest, Decision Tree, Logistic Regression and Bagging Classifier, to train and test the models.
4. Model discussion: The study evaluates the performance and accuracy of the models using various metrics and compares them with other studies.

**FIGURE 1:**
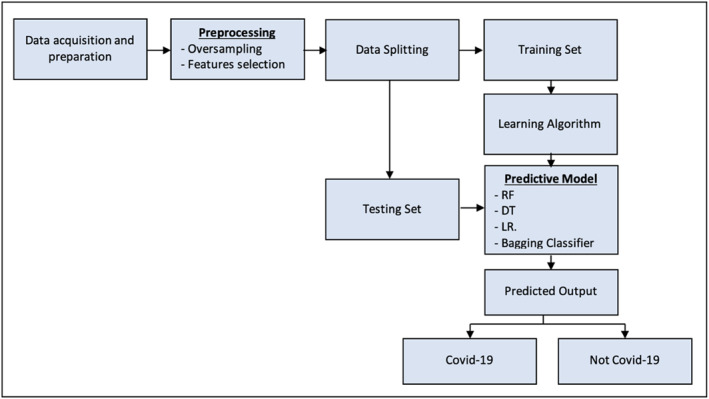
The study flow chart.

Figure 1 shows the proposed workflow used for the classification process in this study.

### 3.1 Data acquisition

The primary objective of this study was to develop an enhanced model that can be used to predict COVID‐19 from data of patients presenting to the Emergency Room with Acute Respiratory Symptoms. The following section provides detailed information about our dataset.

#### 3.1.1 Description of the dataset

The dataset comprises of patients aged over 14 years who presented to the Emergency Department (ED) at two hospitals in Riyadh city in Saudi Arabia namely the Security Forces Hospital (SFH) and the King Fahad Medical City (KFMC) with acute respiratory symptoms between 01 January 2021 and 28 February 2022. 635 patients were drawn from the SFH and 280 were from KFMC hospital. The dataset from each of the two selected hospitals consisted of the following information: Demographics, respiratory symptoms, comorbidities, CXR images with CXR information, Laboratory work, Physician's final diagnosis and PCR test results. The demographics data included Study ID, Unique medical record number (MR), gender, age and smoking status. The respiratory symptoms data included cough, SOB, chest pain, haemoptysis and other respiratory symptoms. The comorbidities data consisted of obesity, DM, HTN, chronic lung disease, CAD, heart failure, CVA, immunosuppression, renal failure, liver failure, malignancy and other comorbidities. The radiographic data included date of CXR, CXR view, CXR image, CXR findings distribution, CXR findings patterns, and radiologist CXR diagnosis. The laboratory work comprised of WBC, PLT, CRP, D. Dimer, Ferritin, and the COVID‐19 PCR test result. The Physician's final diagnosis was indicated by the final diagnosis of the ER consultation team if the patient was discharged, or the admitting team if the patient was admitted. We collected the same features with similar conditions from both hospitals. The two datasets from the two hospitals with all their information were merged into one dataset which consists of one excel file with four sheets for the four different categories as stated below: 1). 89 patients with no chronic lung conditions and without COVID‐19, 2). 296 patients with no chronic lung conditions but with COVID‐19, 3). 51 patients with chronic lung conditions and without COVID‐19, and lastly but not least, 4). 242 patients with chronic lung conditions and with COVID‐19. All these data were analysed with the view to identifying an appropriate model that can be used to predict and detect COVID‐19. The dataset comprised of a total of 538 cases of COVID‐19 and 140 non‐COVID‐19 cases. Using these data, we designed a model that can be used to predict COVID‐19 based on these features. The code and data used in this study are publicly available on GitHub. They can be accessed at the following link: https://github.com/malfhdan/alfhdan.git. This repository contains all the necessary scripts and datasets to reproduce the results presented in this paper. Users are encouraged to review the README file for detailed instructions on how to utilise the provided resources. Table 2 presents details of all the dataset features that were used by the developed model.

**TABLE 2:** Dataset features.

A. Demographics	Study ID (For COVID patients: starts with 100,001/For non‐COVID patients: starts with 200,001)
	MRN
	Gender (male/female)
	Age (14 years +)
	Smoking status (smoker/non‐smoker/X‐smoker)
B. Respiratory symptoms	Cough (present/absent)
	SOB (present/absent)
	Chest pain (present/absent)
	Haemoptysis (present/absent)
	Other respiratory symptoms (present/absent)
C. Comorbidities	Obesity (present/absent)
	DM ‐ Diabetes Mellitus (present/absent)
	HTN ‐ Hypertension (present/absent)
	Chronic lung disease (absent/COPD/Asthma/ILD/Bronchiectasis/lung cancer/Pulmonary hypertension/others)
	CAD ‐ Coronary Artery Disease (present/absent)
	Heart failure (present/absent)
	CVA Cerebral vascular Accident (present/absent)
	Immunosuppression (present/absent)
	Renal failure (present/absent)
	Liver failure (present/absent)
	Malignancy (present/absent)
	Other comorbidities (present/absent)
D. Information about image	Date of new CXR (dd‐mm‐yyyy)
	CXR view (Posteroanterior (PA): is frequently used to aid in diagnosing a range of acute and chronic conditions involving all organs of the thoracic cavity/Anteroposterior (AP): is an alternative to the PA view when the patient is too unwell to tolerate standing or leaving the bed
	CXR image
	CXR findings distribution‐1 (Unilateral/Bilateral)
	CXR findings distribution‐2
	1 = upper zone that is, below the clavicles and above the cardiac silhouette
	2 = mid zone that is, the level of the hilar structures
	3 = lower zone that is, the lung bases
	4 = patchy
	5 = diffuse
	CXR findings patterns
	1 = alveolar opacity that is, fluffy infiltrate, like the one seen in pneumonia and heart failure
	2 = reticular opacity that is, linear infiltrate, like the one seen in ILD
	3 = pulmonary nodule/nodules or mass/masses.
	Radiologist CXR diagnosis: Radiologist diagnosis
	0 = normal CXR
	1 = Pneumonia
	2 = COVID‐19 infection
	3 = aspiration pneumonia
	4 = Heart failure/Fluid overload
	5 = Asthma/COPD
	6 = bronchiectasis
	7 = ILD
	8 = lung cancer
	9 = others
E. Lab test	WBC ‐ White Blood cells count (initial lab result)
	PLT ‐ Platelet (initial lab result)
	CRP ‐ C‐Reactive Protein (initial lab result)
	D. Dimer (initial lab result)
	Ferritin (initial lab result)
F. COVID PCR test	Date (dd‐mm‐yyyy)
	COVID PCR result (negative/positive)
G. Diagnosis	Final diagnosis of the ER/consultation team (if discharged) or admitting team (if admitted)
	Physician final diagnosis (Pneumonia/COVID‐19 infection/Aspiration pneumonia/Heart failure/Fluid overload/CAD/Asthma/COPD/bronchiectasis/ILD/Lung cancer/others)

#### 3.1.2 Data analysis and visualisation

**FIGURE 2:**
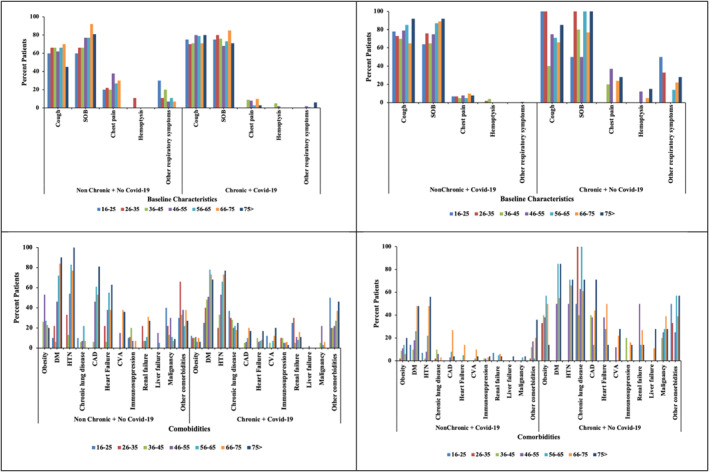
Graphical visualisation of baseline characteristics.

**FIGURE 3:**
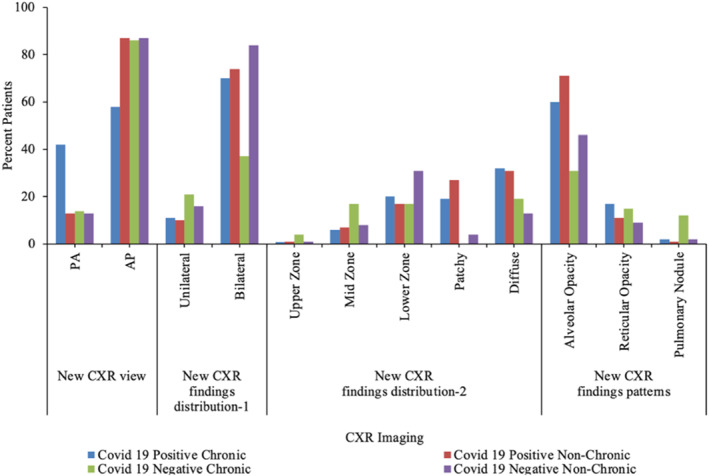
Graphical visualisation of new current imaging data.

Based on the demographical data, it was found that the average age of the patients across the four groups of the disease was non‐significantly different (Table 3). There were more males (58%) among the patients with chronic lung condition and positive COVID‐19; however, the proportion of males was lowest (27%) among the patients who had chronic lung condition but who tested negative for COVID‐19 (Table 3). It was also shown that most of the patients were non‐smokers amongst all the four disease categories. This was followed by the current smokers who had a relatively higher percentage (14%) among patients with chronic symptoms but with negative results for COVID‐19. In terms of the respiratory symptoms, more patients presented higher percentage in symptoms such as cough, SOB and chest pains across all the four categories (Table 3 and Figure 2). Some of the patients had AP CXR. In the CXR findings distribution, more patients fell in the bilateral and diffuse category. Results from the new CXR findings pattern showed that aveolar opacity featured in more patients followed by reticular opacity (Table 4 and Figure 3).

**TABLE 3:** Base line characteristics for the patients with or without COVID‐19 infection.

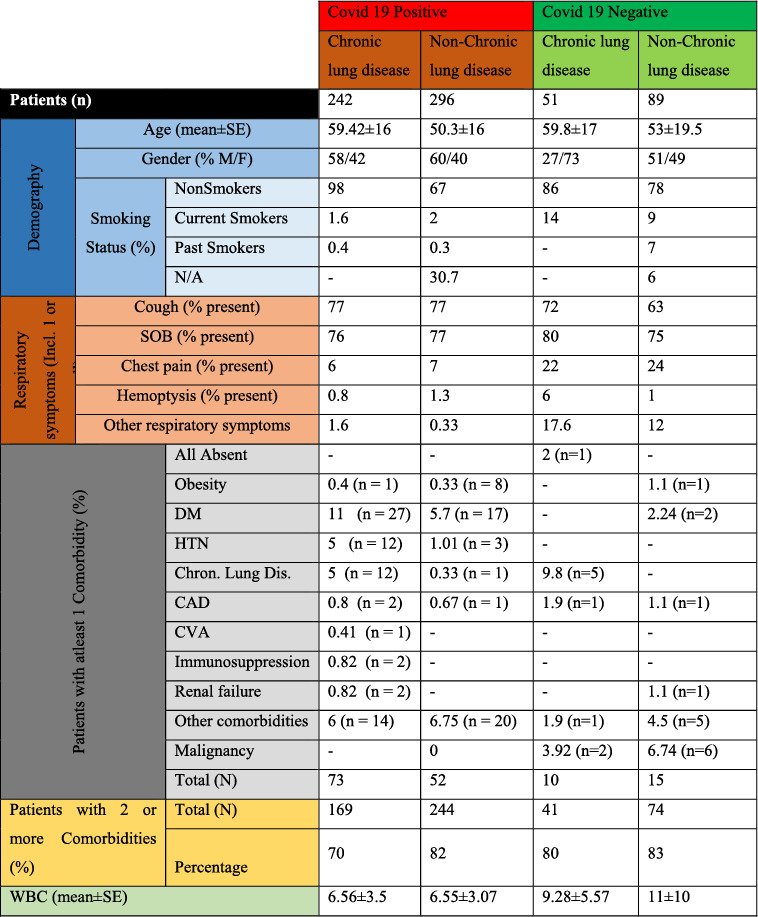

**TABLE 4:** Current Imaging data set for the patients with or without COVID‐19 infection.

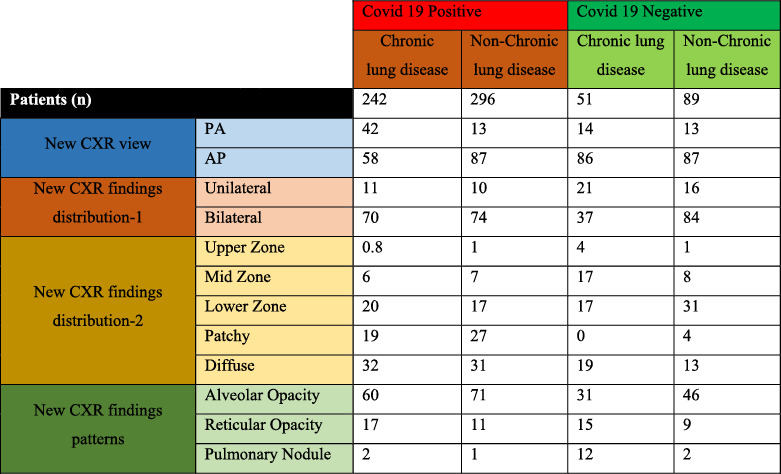

### 3.2 Preprocessing

For effective functioning of machine learning, huge amounts of data are required, including features and variables to make predictions and arrive at highly accurate results. However, if the dataset is used without pre‐processing, this will generate worse off prediction results. The two techniques used for pre‐processing the dataset with the view to improving the accuracy of the models in our study are discussed below.

#### 3.2.1 Oversampling

**FIGURE 4:**
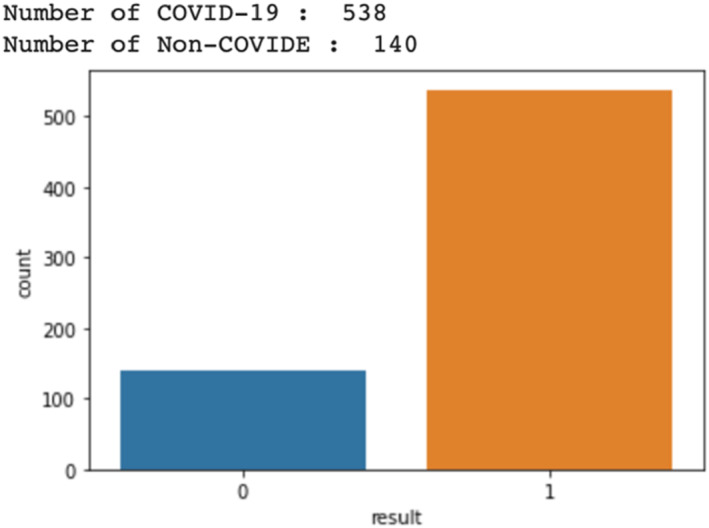
The total number of COVID‐19 and non‐COVID‐19.

One of the well‐researched problems in the machine learning framework is the imbalance of classification [30, 31]. The imbalance classification problem can be defined as a binary classification problem where two class sizes are different making it difficult to make accurate predictions. For instance, when the size of a class which normally provides the most important concept to predict is much lower than the other class, this generates an imbalance problem. In such cases, standard classification learning algorithms may produce biased results in favour of the majority class cases. To address such a problem, the commonly used approach involves applying a pre‐processing stage for rebalancing the training data. This can be achieved by either under sampling, that is, getting rid of the majority class instances or oversampling, which involves adding new minority class instances. Under sampling might result in loss of relevant instances, hence, oversampling is the preferred approach [32]. In addition, this approach is meant to strengthen the concept represented by the minority class, this way, the learning algorithm will be supported to avoid misclassifying the relevant examples. In our study, we had a typical case of imbalanced classification with the number of COVID‐19 being 538 and the number of non‐COVID‐19 being 140. As a result, we made use of the Random Over Sampler function in IMBLearn Library in the python language to increase the non‐COVID‐19 data (which included patients with chronic lung conditions and patients without any chronic lung conditions) and to balance the data (Figure 4).

#### 3.2.2 Feature selection techniques

In the process of developing the machine learning models, several input variables called features are fed into the models. Each column in our dataset represents a feature. In order to train an optimal model, we ensured that we made use of essential features only. Only a few variables in our dataset were relevant for building the model. Most of the variables were either redundant or irrelevant [33]. The overall performance and accuracy of the model is affected by adding all the variables indiscriminately because the model will learn unimportant patterns from the noise. As a result, it is vitally important to identify and choose the most appropriate features from the data and eliminate all the irrelevant or less important features. This is achieved by using feature selection in machine learning [12, 13].

In this study, we were aware that not all features in the dataset were relevant for the training of the models, hence, it was necessary to use feature selection. This is more important than designing the prediction model. In the first instance, this helps to simplify the model by reducing the number of parameters. In addition, by reducing the training time this also reduced storage and computational costs. At the same time, this reduced the chances of losing important information or the degradation of learning performance. One of the motivating factors is that this helps to produce the best model with high prediction results and minimal errors that facilitate the enhancement of accuracy and the classification performance.

Initially, we started with 36 features from both hospitals, however, after data cleaning we eliminated all the features that did not have adequate data and we were left with 22 features. We used 22 features from the dataset with the four ML algorithms and proceeded to discuss the results of feature selection with the specialist doctors. Based on the advice from the specialist doctors we then selected the top 12 features from the dataset (New CXR findings patterns, New CXR findings distribution‐1, New CXR findings distribution‐2, Smoking status, WBC, Renal failure, Obesity, Chest pain, Chronic lung disease, CAD, Heart Failure and Malignancy) for subsequent training of the supervised ML algorithm. We employed the SelectKBest for the feature selection for use with the supervised models. Table 5 shows the score of the top 12 features that were selected from the 22 features based on the univariate statistical tests. It is worth mentioning that we made a selection of the best predictors for the chosen target variables which consisted of the pattern and distribution of the CXR abnormalities, smoking status, chest pain, WBC count, and specific comorbidities such as renal failure, obesity, chronic lung disease, CAD, heart failure and malignancy. The features selection is a pre‐processing step to an estimator which is important for the removal of noise and less important data, thereby reducing the training time for the models. By using the selected 12 features we were able to improve the performance and accuracy of the models.

**TABLE 5:** The score of the features.

Features name	Score
New CXR findings patterns	14,307.39049
New CXR findings distribution‐1	14,269.08737
New CXR findings distribution‐2	12,310.00455
Smoking status	11,766.62354
WBC	690.74681
Renal failure	590.42172
Obesity	510.02235
Chest pain	240.00304
Chronic lung disease	121.3038
CAD	92.4466
Heart failure	81.33121
Malignancy	75.57143
CVA	21.04348
Liver failure	19.59259
Age	19.08651
HTN	14.33508
Immunosuppression	10.28571
Haemoptysis	8.16667
Gender	6.62727
DM	6.29719
Cough	1.7424
SOB	0.15152

### 3.3 Supervised machine learning models for predicting COVID‐19

Supervised learning is a subcategory of machine learning and artificial intelligence. It is characterised by its use of labelled datasets to train algorithms that are used to classify data or to make accurate prediction of outcomes. When the input data is introduced into the model, it makes adjustments to its weights until the model has been appropriately fitted and this takes place as part of the cross‐validation process. During the training process, the margin of error is minimised with the view to enabling the generalisation of learning to facilitate the prediction of results for the new cases [34]. If the labels are like separate classes, the output is called classification; and is called regression if the output is in continuous quantities. There are several algorithms in each category [35].

Some algorithms are well‐suited for modelling with small dataset. We used small data sets to model some algorithms that are appropriate for this purpose. We train various models that work well with small data and choose the one that has the best performance, the shortest training time, and the most relevant features for specialist doctors to diagnose Covid. In the following section, we describe the machine learning models that we applied to the COVID‐19 dataset. In addition, we did not tune the hyperparameters of the models, instead we used the default hyperparameters of all the models.

#### 3.3.1 Random forest algorithms (RF)

As the name implies, this is a powerful and versatile supervised ML algorithm that grows and combines multiple decision trees to create a “forest” that is used as individual predictors. The way this works is that, instead of trying to develop an optimised method in one goal, several predictors are created and their different predictions are brought together (pooled). In this case, the one that gets the most votes becomes the final predicted class [36]. The main advantage of the random forest algorithm is its ability to be used for both classification and regression problems and this constitutes the bulk of the existing ML systems.

#### 3.3.2 Bagging classifier

A technique for improving the accuracy of predictions made by a supervised learning algorithm is called bagging or bootstrap aggregating [37]. This involves training several different models on different randomly selected subsets of the training data. In the end, the different predictions are combined using some form of voting scheme. One of the celebrated advantages of bagging is that it minimises variance of the predictions made by the supervised learning algorithm without any significant compromises to the accuracy level. As a result, this is a useful technique in situations where the cost of making mistakes is high, for instance, in medical diagnosis. This facilitates a compromise of some accuracy for enhanced robustness.

#### 3.3.3 Decision tree (DT)

This is a supervised learning technique that can be utilised for both classification and regression problems [38]. However, it is mostly used for addressing the classification problems. This tree‐structured classifier's internal nodes represent the features of dataset, branches represent the decision rules and the outcomes are represented by each leaf node. There are two nodes available in a decision tree and these are the decision node and the leaf node. Decision nodes have many different branches and are used for decision making. On the other hand, the leaf nodes do not contain any more branches and are the output of those branches. The features of the existing dataset constitute the basis of the decisions or the tests that are carried out.

#### 3.3.4 Logistics Regression (LR)

This is one of the commonly used machine learning algorithms under the category of supervised learning techniques [39]. It is useful for the prediction of the categorical dependent variable using a particular set of independent variables. In this case, the outcome must be a categorical/discrete value. The plausible options include either Yes or No, 0 or 1, True or False, among others. Rather than giving the exact value as 0 or 1, it gives the probability value which is found between 0 and 1. This is an important machine learning algorithm given that it can produce probabilities and classify new data using both continuous and discrete datasets.

### 3.4 The protocol

Following the application of the oversampling technique to increase the data and to ensure that the datasets were balanced, feature selection was used to select the top 12 from the 22 features in the dataset. The selection of the features was done in collaboration with the doctors in the intensive care unit (ICU) and they agreed on the quality of the selected 12 features. The dataset that emerged from the two hospitals had four different categories and this was divided into two sets including 80% that was used for training and 20% that was used for testing. The training set was used to train four supervised machine learning algorithms for the prediction of COVID‐19 from patients that have chronic lung conditions. After the training, the developed algorithm was then tested using the test set. The enhanced model that was developed was discussed with the doctors from the hospitals and they want to apply this model when dealing with patients in the hospital and plans are underway to publish the case study. As can be gleaned here, this shows the benefit of the study, a model has been produced from the pre‐trained models and this is capable of predicting COVID‐19 from patients suffering from chronic lung conditions. Using the model, it is possible to identify if the patients have got COVID‐19 or not from the different features presented by the patients when they enter the Emergency Department with respiratory symptoms. It is necessary to appreciate that the model has a high performance compared to the existing models discussed in literature, and this has also been developed in collaboration with doctors whose expertise helped to ensure that the quality of the dataset was good for accurate predictions. The next chapter presents a more detailed explanation of the model.

### 3.5 Hyper‐parameters tuning

Fine‐tuning hyperparameters is a crucial step in the development of machine learning models, as it involves determining the optimal settings that lead to highest model performance. These settings are critical as they influence the precision and efficiency of the model in tasks such as prediction and classification. Enhanced model performance, achieved through meticulous hyperparameter optimisation, translates to better learning from data and more dependable outcomes. The essence of this process is to unlock the full potential of the model while significantly reducing errors. The GridSearchCV algorithm, part of the scikit‐learn library, is employed for this purpose. It streamlines the search for the best hyperparameter mix, guaranteeing peak model performance. The following Tables 6, 7, 8, 9 showcase the original and fine‐tuned hyperparameters across different machine learning models.

**TABLE 6:** Random forest hyper‐parameters.

Hyper‐parameters	Default	Tuning	Range
n_estimators	100	50	Unlimited
max_features	sqrt	log2	“sqrt”, “log2”, none
max_depth	None (nodes are expanded until all leaves are pure)	6	Integer number
max_leaf_nodes	None (Unlimited number of leaf nodes)	9	Integer number

**TABLE 7:** Decision tree hyper‐parameters.

Hyper‐parameters	Default	Tuning	Range
max_depth	None (nodes are expanded until all leaves are pure)	30	Integer
min_samples_split	2	2	Integer or float
min_samples_leaf	1	1	Integer or float

**TABLE 8:** Bagging classifier hyper‐parameters.

Hyper‐parameters	Default	Tuning	Range
n_estimators	10	50	Unlimited
max_samples	1	0.8	Integer or Float
max_features	1	0.6	Integer or Float

**TABLE 9:** Logistic regression hyper‐parameters.

Hyper‐parameters	Default	Tuning	Range
multi_class	auto	Multinomial	‘auto’, ‘ovr’, ‘multinomial’
Solver	lbfgs	Newton‐cholesky	‘lbfgs’, ‘liblinear’, ‘Newton‐cg’, ‘Newton‐cholesky’, ‘sag’, ‘saga’
Penalty	l2	l2	‘l1’, ‘l2’, ‘elasticnet’, none
Tol	0.0001	0.01	0–1

## 4 EVALUATION METRICS AND RESULTS

The evaluation of machine learning models or algorithms is significantly important in any project. There exist different types of evaluation metrics that can be used to test a developed model. This section presents details of the evaluation metrics used to measure the developed machine learning model in this study. For the measurement of the performance of the ML classification model, the confusion matrix can be used. However, it must be borne in mind that the confusion matrix itself is not considered a metric. Basically, it is a table which consists of two dimensions showing actual values and the predicted values. For example, researchers may want to design a classifier that helps to diagnose patients as either COVID‐19 or non‐COVID‐19. In this study, each classification can be one of the four outcomes on the basis of how it matches up to the actual value:

- True Positives (TP): The cases in which we predicted COVID‐19 and the actual output was COVID‐19.
- True Negatives (TN): The cases in which we predicted non‐COVID‐19 and the actual output was non‐COVID‐19.
- False Positives (FP): The cases in which we predicted COVID‐19 and the actual output was non‐COVID‐19.
- False Negatives (FN): The cases in which we predicted non‐COVID‐19 and the actual output was COVID‐19.

## 5 EXPERIMENTAL RESULTS

**FIGURE 5:**
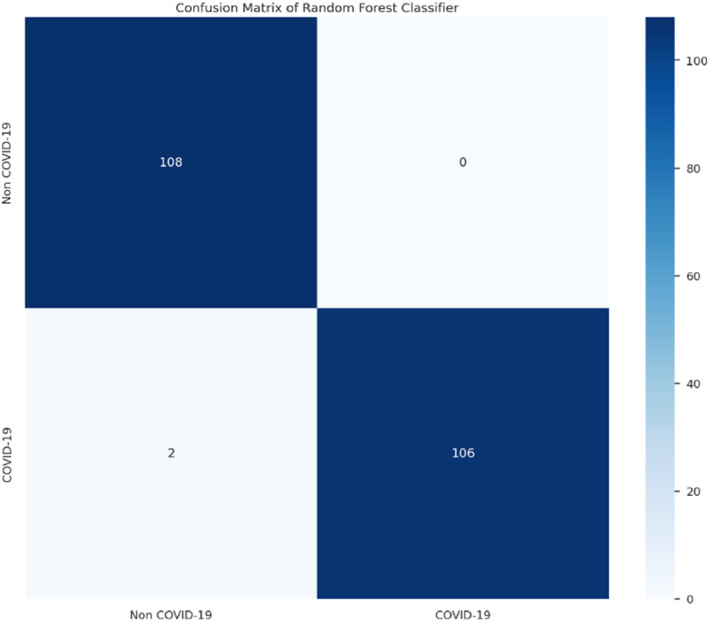
Confusion matrix of random forest.

**FIGURE 6:**
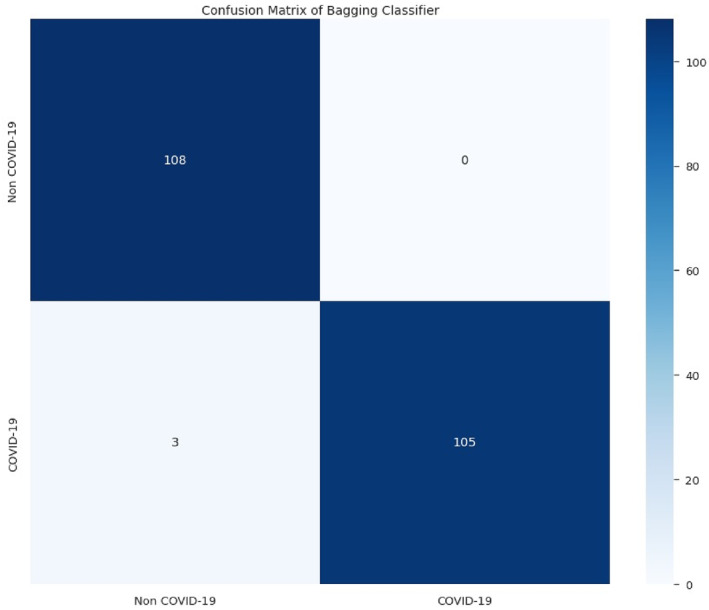
Confusion matrix of bagging classifier.

**FIGURE 7:**
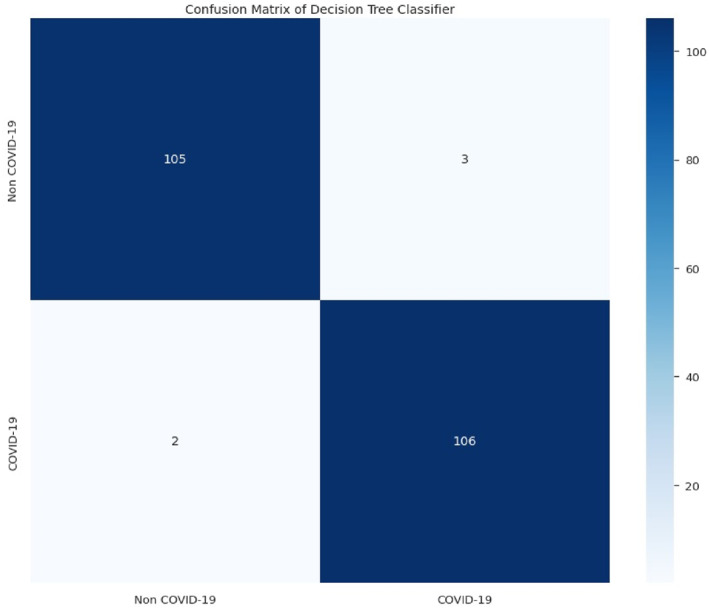
Confusion matrix of decision tree classifier.

**FIGURE 8:**
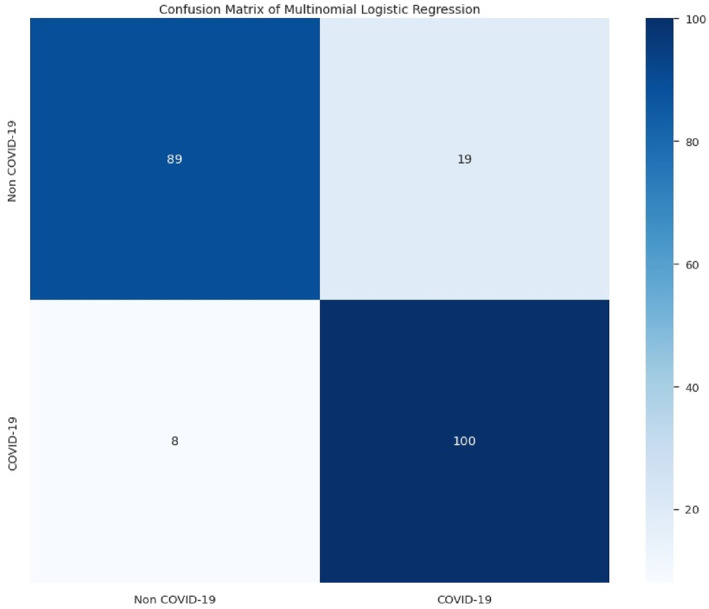
Confusion matrix of multinomial logistic regression.

The following results in Figures 5, 6, 7, 8 were obtained when the developed algorithms were tested on the test set.

From the above Figures 5, 6, 7, 8, the values of TP, TN, FP and FN were drawn, and these were used to compute the performance of the algorithms as shown in Table 10 below.

**TABLE 10:** Confusion matrix metrics of algorithms.

Models	TP	TN	FP	FN
Random forest	108	106	2	0
Bagging classifier	108	105	3	0
Decision tree	105	106	2	3
Multinomial logistic regression	89	100	8	19

From Table 10, the most important metrics used to measure the performance of machine learning algorithms were calculated as shown in Table 11.

**TABLE 11:** Evaluation metrics of all algorithms.

Models	Accuracy %	Specificity %	Precision %	Recall %	F1‐score %
Random forest	99.07	98.15	98.18	1	99
Bagging classifier	98.61	97.22	97.28	100	98.63
Decision tree	97.69	98.15	98.13	97.22	97.67
Multinomial logistic regression	87.50	92.59	91.75	82.41	86.83

## 6 IMPORTANCE OF 10‐FOLD CROSS‐VALIDATION AND RESULTS

Ten‐fold cross‐validation is an important method in machine learning to assess a model's efficacy. The approach involves dividing the data into 10 segments, or 'folds', training the model on nine and testing on one. Rotating the test fold through each iteration, this cycle is conducted 10 times. Averaging results from these cycles yields a comprehensive performance evaluation, offering a more reliable measure than a solitary training and testing division. In our study, we employed the 10‐fold cross‐validation technique to assess the performance of our machine learning models. We applied cross‐validation in two distinct approaches as discussed below:

### 6.1 Full dataset oversampling

The first approach involved applying oversampling to the entire dataset before performing cross‐validation. This ensured that both the training and testing sets were balanced in terms of class distribution. The results obtained from this technique are summarised in the following Table 12:

**TABLE 12:** 10‐Fold cross‐validation results for various machine learning models.

Fold model	Random forest	Decision tree	Bagging classifier	Logistic regression
1‐Fold	91.67	89.81	86.11	75
2‐Fold	92.59	90.74	91.67	88.89
3‐Fold	98.15	96.30	95.37	92.59
4‐Fold	97.22	96.30	95.37	81.48
5‐Fold	100	99.07	99.07	91.67
6‐Fold	90.74	93.52	87.96	77.78
7‐Fold	95.33	88.79	91.59	80.37
8‐Fold	95.33	92.52	91.59	87.85
9‐Fold	100	97.20	100	89.72
10‐Fold	99.07	90.65	96.26	92.52
Mean accuracy	96	93	93	86
Standard Deviation	0.03	0.03	0.04	0.06

his approach ensures a thorough assessment of model efficacy, providing a dependable evaluation by considering the variability in training and testing datasets over numerous cycles. The findings indicate that the Random Forest model outperformed others, achieving a high average accuracy rate of 0.96 and a minimal standard deviation of 0.03, reflecting its precision and stability. Both the Decision Tree and Bagging Classifier models showed commendable performance with an average accuracy of 0.93, though they exhibited slightly higher fluctuations (standard deviations of 0.03 and 0.04, respectively). The Logistic Regression model, however, recorded the lowest average accuracy at 0.86 and the greatest standard deviation of 0.06, suggesting more pronounced inconsistencies in performance. Overall, the Random Forest model emerged as the most accurate and consistent, making it the preferred choice for this dataset.

### 6.2 Training set oversampling

The second approach applied oversampling to the training set only. This method maintained a balanced training set while keeping the testing set unaltered, providing a more realistic evaluation of the model's performance on imbalanced data. The dataset was divided into two subsets: 80% was allocated for training and 20% for testing. After training the models and evaluating their performance on the test set, the results summarised in Table 13 were obtained.

**TABLE 13:** Accuracy of the proposed models.

Model	Accuracy %
Random forest classifier	90.44
Decision tree	88.24
Bagging classifier	89.71
Logistic regression	91.91

Subsequently, a 10‐fold cross‐validation technique was employed to further train and evaluate the models using the entire dataset. The results of this process are presented in Table 14.

**TABLE 14:** 10‐Fold cross‐validation accuracy.

Fold model	Random forest	Decision tree	Bagging classifier	Logistic regression
1‐Fold	76. 74	73.53	75.00	80.88
2‐Fold	88.23	89.71	91.18	86.76
3‐Fold	92.64	86.76	89.70	88.24
4‐Fold	91.18	83.82	91.18	94.11
5‐Fold	94.12	86.76	92.65	92.65
6‐Fold	85.30	77.94	83.82	82.35
7‐Fold	89.70	83.82	91.18	88.23
8‐Fold	88.23	82.35	88.23	92.65
9‐Fold	94.03	97.01	95.52	92.54
10‐Fold	92.54	82.10	92.54	95.52
Mean accuracy	89.24	84.38	89.10	89.39
Standard Deviation	0.05	0.06	0.05	0.04

To optimise the model performance (refer to Tables 15, 16, 17, 18 for the hyperparameters), hyperparameter tuning was conducted exclusively on the training set. The oversampling technique was applied to the training set to balance the dataset by increasing the representation of the non‐COVID class. The GridSearchCV algorithm from the scikit‐learn library was utilised to determine the optimal hyperparameters that yield the best results.

**TABLE 15:** Random forest hyperparameters.

Hyper‐parameters	Default	Tuning	Range
n_estimators	100	25	Unlimited
max_features	sqrt	log2	“sqrt”, “log2”, none
max_depth	None (nodes are expanded until all leaves are pure)	9	Integer number
max_leaf_nodes	None (Unlimited number of leaf nodes)	32	Integer number

**TABLE 16:** Decision tree hyperparameters.

Hyper‐parameters	Default	Tuning	Range
Criterion	gini	Entropy	“gini”, “entropy”, “log_loss”
ccp_alpha	0.0	0.01	0–1
Splitter	Best	Random	“Best”, “random”

**TABLE 17:** Bagging classifier hyperparameters.

Hyper‐parameters	Default	Tuning	Range
n_estimators	10	40	Unlimited
max_samples	1	0.8	Integer or Float
max_features	1	0.7	Integer or Float

**TABLE 18:** Logistic regression hyperparameters.

Hyper‐parameters	Default	Tuning	Range
multi_class	auto	Multinomial	‘auto’, ‘ovr’, ‘multinomial’
Solver	lbfgs	Newton‐cholesky	‘lbfgs’, ‘liblinear’, ‘Newton‐cg’, ‘Newton‐cholesky’, ‘sag’, ‘saga’
Penalty	l2	None	‘l1’, ‘l2’, ‘elasticnet’, none
C	1.0	0.1	0–1

**FIGURE 9:**
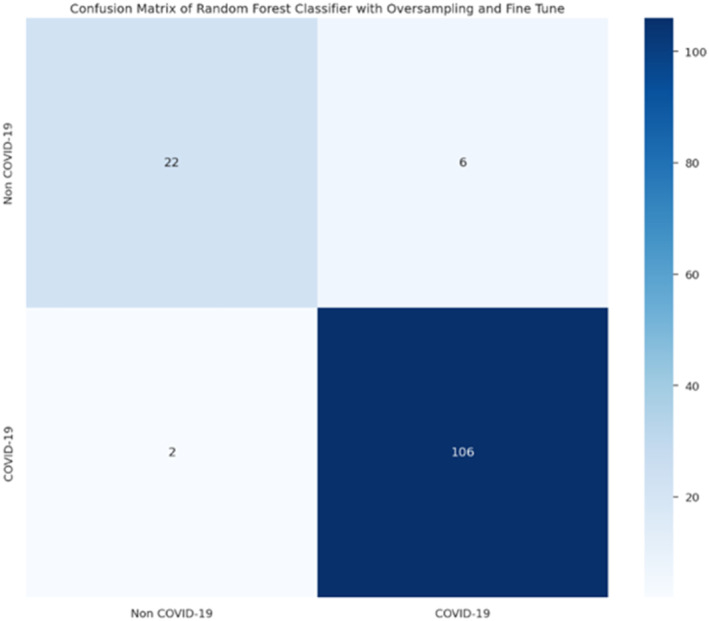
Confusion matrix of random forest.

**FIGURE 10:**
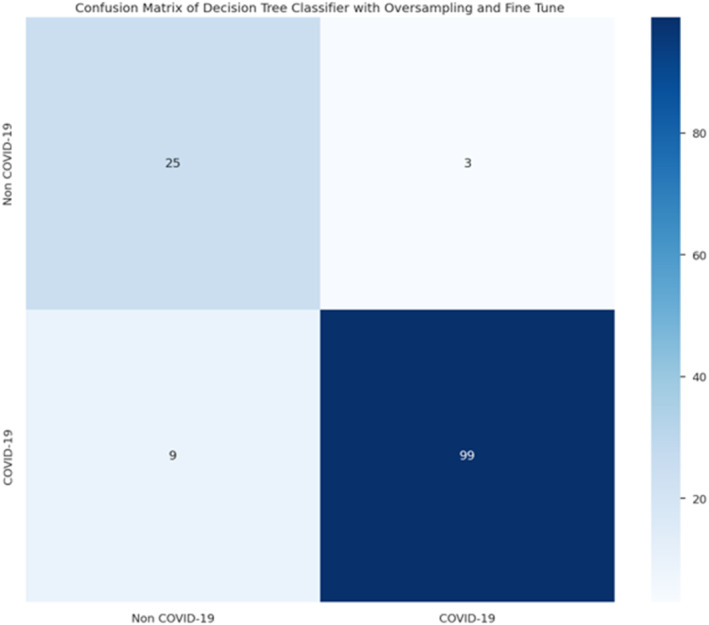
Confusion matrix of decision tree.

**FIGURE 11:**
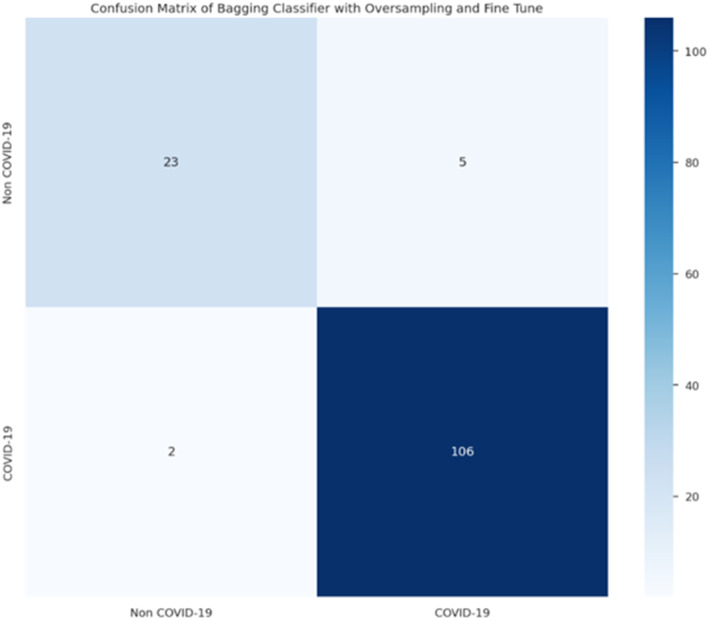
Confusion matrix of bagging classifier.

**FIGURE 12:**
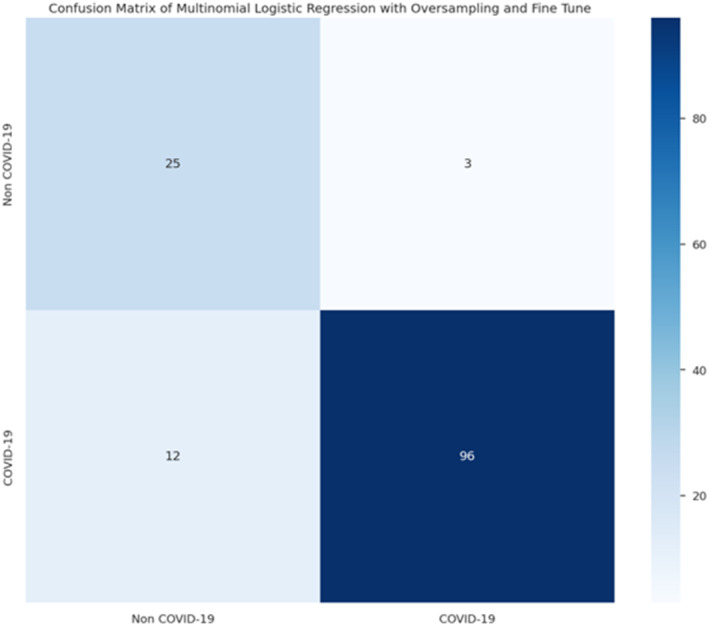
Confusion matrix of logistic regression.

After refining and training the proposed models, their performance was evaluated using confusion matrices, as shown in the results as illustrated in Figures 9, 10, 11, 12.

From these confusion matrices, the following performance metrics were calculated: accuracy, sensitivity, and specificity as shown in Table 19 below.

**TABLE 19:** Performance metrics.

Metric	Models			
	Random forest	Decision tree	Bagging classifier	Logistic regression
Accuracy %	94.12	91.18	94.85	88.97
Sensitivity %	78.57	89.29	82.14	89.29
Specificity %	98.15	91.67	98.15	88.89

After refining and training the proposed models, their performance was evaluated using confusion matrices. By comparing the results from these two approaches, we gained insights into how oversampling and fine‐tuning hyperparameter techniques impact the accuracy, sensitivity, and specificity of the proposed models. Notably, the models achieved high performance even with the imbalanced and limited dataset.

## 7 STATISTICAL SIGNIFICANCE OF THE RESULTS

To determine the statistical significance of the differences in performance between the models, we conducted paired *t*‐tests for each pair of models. The results are summarised in the following Table 20:

**TABLE 20:** Statistical significance of model performance comparisons.

Model comparison	t‐statistic	*p*‐value
Random forest versus Decision tree	2.584	0.0295
Random forest versus Bagging classifier	4.832	0.0009
Random forest versus Logistic regression	7.020	0.00006
Decision tree versus Bagging classifier	−0.009	0.993
Decision tree versus Logistic regression	4.101	0.0027
Bagging classifier versus Logistic regression	5.875	0.0002

The statistical analysis indicates that the Random Forest model outperforms the other models. It shows notably better results when compared with the Decision Tree, Bagging Classifier, and Logistic Regression models. Performance metrics for both the Decision Tree and Bagging Classifier are comparable, whereas the Logistic Regression model lags behind the others in terms of effectiveness.

## 8 DISCUSSION

The primary aim of this study was to develop an appropriate approach for the diagnosis of COVID‐19. As a result, data drawn from 915 patients from two hospitals in Saudi Arabia were used to develop the model. The dataset from each of the two selected hospitals consisted of seven excel spreadsheets including the following information: Demographics, respiratory symptoms, comorbidities, CXR images with CXR information, Laboratory test results, and PCR test results. These data initially comprised 36 features. However, after data cleaning, all features that did not have adequate data were eliminated, leaving 22 features. These 22 features were then used in the dataset with four machine learning algorithms, and the results of feature selection were discussed with specialist doctors. Based on their advice, the top 12 features from the dataset were selected.

As Table 11 shows, the random forest, decision tree, bagging classifier, and logistic regression have accuracy rates of 99.07%, 97.69%, 98.61%, and 87.5%, respectively. The random forest bagging classifier outperforms the others because it combines multiple decision trees that are trained on different subsets of the data and averages their predictions to increase the accuracy. The random forest does not depend on a single decision tree but uses the majority vote of all the trees to determine the final output. The more trees in the random forest, the higher the accuracy and the lower the risk of overfitting. The bagging classifier comes in second place because it uses ensemble learning to improve its stability and accuracy. The algorithm is also very effective with datasets containing a small number of samples. In conclusion, data from the same Table 11 indicates that the random forest algorithm has outweighed the performance of the other algorithms in terms of precision, specificity, recall, and F1‐score. It was useful to compare the model developed in our study with other existing models to appreciate its enhanced performance for the prediction of COVID‐19. Table 21 presents the comparative results, which help to demonstrate how the proposed approach from our study outperforms the other existing models.

**TABLE 21:** Comparative Analysis between the new approach and some existing models.

Study	Features	Techniques	Performance (%)
Yao et al. [24]	Clinical information and blood/urine test data	SVM	Accuracy = 81.48
			Sensitivity = 83.33
			Specificity = 100
Brinati et al. [25]	Routine blood exams (white blood cells counts, and the platelets, CRP, AST, ALT, GGT, ALP, LDH plasma levels)	‐Logistic regression	Accuracy 86
		‐ Random forest	Sensitivity 95
Yang et al. [26]	Demographic features with routine laboratory	‐ Logistic regression	Sensitivity = 76.1
		‐ Decision tree	Specificity = 80.8
		‐ Random forest	AUC = 85.40
		‐ Gradient boosting decision tree (GBDT)	
Hu et al. [27]	Demographic, clinical and first laboratory findings	‐ Logistic regression	Sensitivity = 89.2
		‐ Random forest	Specificity = 68.70
		‐ PLSR	AUC = 89.20
		‐ Elastic net	
		‐ BFDA	
Khanday et al. [28]	Clinical reports	‐ Logistic regression	Precision = 94
		‐ Multinomial Naaive Bayesian	Recall = 96
			F1‐score = 95
			Accuracy = 96.2%.
Sun et al. [29]	Clinical and laboratory features	Support vector machine	Accuracy: 77.5%
			Specificity: 78.4%
Our Proposed method	‐ Demographics: (Gender, age, Smoking status)	Random forest	Single train test split accuracy:
	‐ Respiratory symptoms: (Cough, SOB, chest pain, Haemoptysis,	Decision tree	Random forest = 99.07%
	Other respiratory symptoms)	Bagging classifier	Decision tree = 97.69%
	‐ Comorbidities: (Obesity, DM, HTN, chronic lung disease, CAD, Heart failure, CVA, immunosuppression, renal failure, liver failure, malignancy, Other comorbidities)	Multinomial logistic regression	Bagging classifier = 98.61%
	‐ X‐ray information: (CXR view, CXR image, CXR findings distribution‐1, CXR findings distribution‐2, CXR findings patterns, radiologist CXR diagnosis)		Multinomial logistic regression = 87.50%
	‐ Lab test: (CRP, D. Dimer, Ferritin)		Accuracy for 10‐fold cross‐validation (full Dataset Oversampling):
			Random forest = 100%
			Decision tree = 99%
			Bagging classifier = 100%
			Multinomial logistic regression = 92%
			Accuracy for 10‐fold cross‐validation (training Set Oversampling):
			Random forest = 94.12%
			Decision tree = 91.18%
			Bagging classifier = 94.85%
			Multinomial logistic regression = 88.89%

During discussions with the specialist doctors, it was observed that the pattern and distribution of the Chest X‐Ray lung abnormalities are the most relevant features when it comes to differentiating COVID‐19 from non‐COVID‐19 conditions. Usually, COVID‐19 infection presents radiologically with bilateral airspace infiltrates in a peripheral and lower zone predominant distribution.

## 9 CONCLUSION

The deadly coronavirus (COVID‐19) was first detected in Wuhan, China in December 2019. The highly infectious virus spread quickly across the globe causing untold suffering in both healthcare systems and in the global economies. It has been shown that the quick detection of COVID‐19 patients is essential to stop its spread and its deleterious effects. Thankfully, the ongoing developments in computer algorithms, in particular, artificial intelligence, provides a mechanism to detect the virus quickly in its initial stages thereby facilitating speedy recovery.

Several studies have been conducted focussing on the development of ML and DL models that can be used to detect and predict COVID‐19. However, we are aware that none of the existing models is without its weaknesses due to the size and quality of datasets used, among other factors [6, 7, 8]. It is against this backdrop that the current study was conducted. The primary aim of our study was to develop models with enhanced performance for the diagnosis and prediction of COVID‐19.

This study focused on the prediction of COVID‐19 from patients with acute respiratory symptoms. The study involved working with a dataset comprising of 678 samples of people with COVID‐19 and people without COVID‐19 infection. We developed 4 ML algorithms including Decision Tree, Bagging Classifier and the Logistic Regression. Two techniques namely random oversampling and feature selection were used to address the dataset imbalance classification problem as well as to address the overfitting problem to achieve high performance results. The developed algorithms were trained using 80% of the data and then tested on 20% of the data. The test results revealed that the Bagging classifier had the highest results (98.61% accuracy). The results indicate the power of the ML algorithm in terms of predicting the occurrence of COVID‐19 infection using medical analysis data. Our model predictive variables, which included the pattern and distribution of the CXR abnormalities, smoking status, chest pain, WBC count, and specific comorbidities are readily available in most medical centres; hence, our algorithm can be easily adopted in routine clinical practice.

In consultation with specialist doctors, it was noted that the most critical factors in distinguishing COVID‐19 from other conditions are the pattern and distribution of lung abnormalities observed in Chest X‐Rays. Typically, COVID‐19 manifests radiologically with bilateral airspace infiltrates, which are primarily located in the peripheral and lower zones of the lungs.

Based on findings from this study, we strongly recommend the use of ML algorithms to develop medical diagnostic platforms based on medical analyses. We also recommend generating more data which should be made available to researchers for further studies.

## AUTHOR CONTRIBUTIONS

**Maha Mesfer Alghamdi**: Data curation; investigation; writing ‐ original draft; writing ‐ review & editing. **Naael H. Alazwary**: Conceptualisation; investigation; writing ‐ original draft; writing ‐ review & editing. **Waleed A. Alsowayan**: Formal analysis; investigation; methodology; writing ‐ review & editing. **Mohmmed Algamdi**: Conceptualisation; data curation; investigation; writing ‐ original draft. **Ahmed F. Alohali**: Conceptualisation; formal analysis; investigation; writing ‐ review & editing. **Mustafa A. Yasawy**: Data curation; investigation; methodology; writing ‐ review & editing. **Shatha S. Alghamdi**: Data curation; investigation; methodology; writing ‐ original draft. **Hussein A. Aljaziri**: Conceptualisation; formal analysis; investigation; methodology. **Norah A. Bin Magbel**: Data curation; formal analysis; investigation; writing ‐ original draft. **Nashaat K. Neyazi**: Formal analysis; investigation; writing ‐ original draft; writing ‐ review & editing. **Abeer M. Alghamdi**: Conceptualisation; investigation; writing ‐ original draft; writing ‐ review & editing. **Nujoud H. Almoqati**: Data curation; formal analysis; investigation; writing ‐ review & editing. **Mona M. Alghamdi**: Formal analysis; investigation; methodology; writing ‐ review & editing. **Abdullah M. Alassaf**: Conceptualisation; data curation; investigation; writing ‐ original draft. **Mohammed R. Alshehri**: Conceptualisation; investigation; writing ‐ original draft; writing ‐ review & editing. **Tareq A. AlMazeedi**: Conceptualisation; investigation; methodology; writing ‐ review & editing. **Mohammed N. Alazwary**: Investigation; methodology; writing ‐ original draft; writing ‐ review & editing.

## CONFLICT OF INTEREST STATEMENT

No conflict of interest to declare.

## Data Availability

Data not accessible at this stage due to adherence to ethics.

## References

[1] Roosa, K. , et al.: Real‐time forecasts of the COVID‐19 epidemic in China from february 5th to february 24th, 2020. Infectious disease modelling 5, 256–263 (2020). 10.1016/j.idm.2020.02.002 32110742 PMC7033348

[2] Yan, R. , et al.: Structural basis for the recognition of SARS‐CoV‐2 by full‐length human ACE2. Science 367(6485), 1444–1448 (2020). 10.1126/science.abb2762 32132184 PMC7164635

[3] Rodriguez, A. , et al.: An agent‐based transmission model of COVID‐19 for reopening policy design. Comput. Biol. Med. 148, 105847 (2022). 10.1016/j.compbiomed.2022.105847 35932728 PMC9293792

[4] Wang, L. , Lin, Z. , Wong, A. : Covid‐net: a tailored deep convolutional neural network design for detection of covid‐19 cases from chest x‐ray images. Sci. Rep. 10(1), 19549 (2020). 10.1038/s41598-020-76550-z 33177550 PMC7658227

[5] Li, Y. , Xia, L. : Coronavirus disease 2019 (COVID‐19): role of chest CT in diagnosis and management. Am. J. Roentgenol. 214(6), 1280–1286 (2020). 10.2214/ajr.20.22954 32130038

[6] Houssein, E.H. , et al.: Hybrid quantum‐classical convolutional neural network model for COVID‐19 prediction using chest X‐ray images. Journal of Computational Design and Engineering 9(2), 343–363 (2022). 10.1093/jcde/qwac003

[7] Houssein, E.H. , Emam, M.M. , Ali, A.A. : An optimized deep learning architecture for breast cancer diagnosis based on improved marine predators algorithm. Neural Comput. Appl. 34(20), 18015–18033 (2022). 10.1007/s00521-022-07445-5 35698722 PMC9175533

[8] Houssein, E.H. , et al.: AN efficient multi‐thresholding based COVID‐19 CT images segmentation approach using an improved equilibrium optimiser. Biomed. Signal Process Control 73(2022), 103401 (2022). 10.1016/j.bspc.2021.103401

[9] Ji, T. , et al.: Detection of COVID‐19: a review of the current literature and future perspectives. Biosens. Bioelectron. 166, 112455 (2020). 10.1016/j.bios.2020.112455 32739797 PMC7371595

[10] Khalifa, N. , et al.: Detection of coronavirus (COVID‐19) associated pneumonia based on generative adversarial networks and a fine‐tuned deep transfer learning model using chest X‐ray dataset. arXiv preprint. arXiv: 2004.01184 (2020)

[11] Bukhari, S.U. , et al.: The diagnostic evaluation of Convolutional Neural Network (CNN) for the assessment of chest X‐ray of patients infected with COVID‐19. medRxiv (2020)

[12] Chen, R.C. , et al.: Selecting critical features for data classification based on machine learning methods. Journal of Big Data 7(1), 1–26 (2020). 10.1186/s40537-020-00327-4

[13] Chen, N. , et al.: Epidemiological and clinical characteristics of 99 cases of 2019 novel coronavirus pneumonia in Wuhan, China: a descriptive study. Lancet 395(10223), 507–513 (2020). 10.1016/s0140-6736(20)30211-7 32007143 PMC7135076

[14] Chudasama, Y.V. , et al.: Impact of COVID‐19 on routine care for chronic diseases: a global survey of views from healthcare professionals. Diabetes & Metab. Syndr. Clin. Res. Rev. 14(5), 965–967 (2020). 10.1016/j.dsx.2020.06.042 PMC7308780 32604016

[15] Dyer, O. : Covid‐19: Pandemic Is Having “Severe” Impact on Non‐communicable Disease Care. WHO survey finds (2020) 10.1136/bmj.m2210 32493728

[16] Haybar, H. , Kazemnia, K. , Rahim, F. : Underlying chronic disease and COVID‐19 infection: a state‐of‐the‐art review. Jundishapur Journal of Chronic Disease Care 9(2), 103452 (2020). 10.5812/jjcdc.103452

[17] Kumar, A. , Nayar, K.R. : COVID 19 and its mental health consequences. J. Ment. Health 30(1), 1–2 (2021). 10.1080/09638237.2020.1757052 32339041

[18] Dubey, S. , et al.: Psychosocial impact of COVID‐19. Diabetes Metabol. Syndr.: Clin. Res. Rev. 14(5), 779–788 (2020). 10.1016/j.dsx.2020.05.035 PMC7255207 32526627

[19] Hajat, C. , Stein, E. : The global burden of multiple chronic conditions: a narrative review. Preventive medicine reports 12, 284–293 (2018). 10.1016/j.pmedr.2018.10.008 30406006 PMC6214883

[20] Cohen, S.P. , et al.: Pain management best practices from multispecialty organizations during the COVID‐19 pandemic and public health crises. Pain Med. 21(7), 1331–1346 (2020). 10.1093/pm/pnaa127 32259247 PMC7184417

[21] Xia, Y. , et al.: Risk of COVID‐19 for patients with cancer. Lancet Oncol. 21(4), e180 (2020). 10.1016/s1470-2045(20)30150-9 32142622 PMC7130057

[22] Richardson, S. , et al.: Presenting characteristics, comorbidities, and outcomes among 5700 patients hospitalized with COVID‐19 in the New York City area. JAMA 323(20), 2052–2059 (2020). 10.1001/jama.2020.6775 32320003 PMC7177629

[23] Shuja, J. , et al.: COVID‐19 open source data sets: a comprehensive survey. Appl. Intell. 51(3), 1296–1325 (2021). 10.1007/s10489-020-01862-6 PMC7503433 34764552

[24] Yao, H. , et al.: Severity detection for the coronavirus disease 2019 (COVID‐19) patients using a machine learning model based on the blood and urine tests. Front. Cell Dev. Biol. 8, 683 (2020). 10.3389/fcell.2020.00683 32850809 PMC7411005

[25] Brinati, D. , et al.: Detection of COVID‐19 infection from routine blood exams with machine learning: a feasibility study. J. Med. Syst. 44(8), 1–2 (2020). 10.1007/s10916-020-01597-4 PMC7326624 32607737

[26] Yang, H.S. , et al.: Routine laboratory blood tests predict SARS‐CoV‐2 infection using machine learning. Clin. Chem. 66(11), 1396–1404 (2020). 10.1093/clinchem/hvaa200 32821907 PMC7499540

[27] Hu, C. , et al.: Early prediction of mortality risk among patients with severe COVID‐19, using machine learning. Int. J. Epidemiol. 49(6), 1918–1929 (2020). 10.1093/ije/dyaa171 PMC7543461 32997743

[28] Khanday, A.M. , et al.: Machine learning based approaches for detecting COVID‐19 using clinical text data. Int. J. Inf. Technol. 12(3), 731–739 (2020). 10.1007/s41870-020-00495-9 32838125 PMC7325639

[29] Sun, L. , et al.: Combination of four clinical indicators predicts the severe/critical symptom of patients infected with COVID‐19. J Clin 128, 104431 (2020). 10.1016/J.JCV.2020.104431 PMC7219384 32442756

[30] Onan, A. : Consensus Clustering‐Based Undersampling Approach to Imbalanced Learning, pp. 14. Hindawi Scientific Programming. 2019 (2019). Article ID: 59011087

[31] Fernández, A. , et al.: An insight into imbalanced big data classification: outcomes and challenges. Complex & Intelligent Systems 3(2), 105–120 (2017). 10.1007/s40747-017-0037-9

[32] Cordón, I. , et al.: Imbalance: oversampling algorithms for imbalanced classification in R. Knowl. Base Syst. 161, 329–341 (2018). 10.1016/j.knosys.2018.07.035

[33] Blum, A.L. , Langley, P. : Selection of relevant features and examples in machine learning. Artif. Intell. 97(1‐2), 245–271 (1997). 10.1016/s0004-3702(97)00063-5

[34] Muhammad, I. , Yan, Z. : Supervised machine learning approaches: a survey. ICTACT Journal on Soft Computing 5(3), 21917 (2015)

[35] Sen, P.C. , Hajra, M. , Ghosh, M. : Supervised classification algorithms in machine learning: a survey and review. In: Emerging Technology in Modelling and Graphics: Proceedings of IEM Graph 2018, pp. 99–111. Springer, Singapore (2020)

[36] Breiman, L. : Random forests. Mach. Learn. 451(45), 5–32 (2001)

[37] Breiman, L. : Bagging predictors. Mach. Learn. 24(2), 123–140 (1996). 10.1007/bf00058655

[38] Brijain, M. , et al.: A survey on decision tree algorithm for classification. Int. J. Eng. Dev. Res. (2014). http://www.ijedr.org. accessed 18 November 2022

[39] Connelly, L. : Logistic regression. Medsurg Nurs. 29(5), 353–354 (2020)

